# Ciguatera in Mexico (1984–2013)

**DOI:** 10.3390/md17010013

**Published:** 2018-12-28

**Authors:** Erick J. Núñez-Vázquez, Antonio Almazán-Becerril, David J. López-Cortés, Alejandra Heredia-Tapia, Francisco E. Hernández-Sandoval, Christine J. Band-Schmidt, José J. Bustillos-Guzmán, Ismael Gárate-Lizárraga, Ernesto García-Mendoza, Cesar A. Salinas-Zavala, Amaury Cordero-Tapia

**Affiliations:** 1 Centro de Investigaciones Biológicas del Noroeste S.C. (CIBNOR), A. P. 128., La Paz C. P. 23096, Mexico; dlopez04@cibnor.mx (D.J.L.-C.); fhernan04@cibnor.mx (F.E.H.-S.); jose04@cibnor.mx (J.J.B.-G.); csalinas@cibnor.mx (C.A.S.-Z.); acordero@cibnor.mx (A.C.-T.); 2 Investigación para la Conservación y el Desarrollo A. C. (INCODE), Nayarit 1325-A, Las Garzas, La Paz C. P. 23079, Mexico; herediatap@gmail.com; 3 Centro de Investigación Científica de Yucatán (CICY), Unidad de Ciencias del Agua, (UCIA), Calle 8, No. 39, Mz. 29, S. M. 64, Cancún C. P. 77524, Mexico; almazan@cicy.mx; 4 Instituto Politécnico Nacional, Centro Interdisciplinario de Ciencias Marinas (IPN-CICIMAR), Av. IPN s/n, Playa Palo de Santa Rita, La Paz C. P. 23096, Mexico; cbands@ipn.mx (C.J.B.-S.); igarate@ipn.mx (I.G.-L.); 5 Centro de Investigación y Científica y de Educación Superior de Ensenada (CICESE), Carretera Ensenada-Tijuana 3918, Zona Playitas, Ensenada C. P. 22860, Mexico; ergarcia@cicese.mx

**Keywords:** Ciguatera fish poisoning, Mexico, epidemiological, treatment, marine toxins, ciguatoxins, ecological risk, *Gambierdiscus*

## Abstract

Historical records of ciguatera in Mexico date back to 1862. This review, including references and epidemiological reports, documents 464 cases during 25 events from 1984 to 2013: 240 (51.72%) in Baja California Sur, 163 (35.12%) in Quintana Roo, 45 (9.69%) in Yucatan, and 16 (3.44%) cases of Mexican tourists intoxicated in Cuba. Carnivorous fish, such as snapper (*Lutjanus*) and grouper (*Epinephelus* and *Mycteroperca*) in the Pacific Ocean, and great barracuda (*Sphyraena barracuda*) and snapper (*Lutjanus*) in the Atlantic (Gulf of Mexico and Caribbean Sea), were involved in all cases. In the Mexican Caribbean, a sub-record of ciguatera cases that occurred before 1984 exists. However, the number of intoxications has increased in recent years, and this food poisoning is poorly studied in the region. Current records suggest that ciguatera fish poisoning in humans is the second most prevalent form of seafood poisoning in Mexico, only exceeded by paralytic shellfish poisoning (505 cases, 21 fatalities in the same 34-year period). In this study, the status of ciguatera in Mexico (epidemiological and treatment), and the fish vectors are reviewed. Dinoflagellate species *Gambierdiscus*, *Ostreopsis*, and *Prorocentrum* are related with the reported outbreaks, marine toxins, ecological risk, and the potential toxicological impact.

## 1. Introduction

Ciguatera fish poisoning (CFP) is a food-borne illness endemic to tropical and subtropical waters. Numerous benthic and pelagic fishes accumulate the dinoflagellate’s ladder frame polyether toxins, ciguatoxins (CTXs), in their flesh and viscera [1,2]. In tropical regions, CFP is a serious problem impacting both human health and the fishing industry. More than 50,000 CFP cases are reported annually, which is the most commonly occurring cause of human poisoning through seafood consumption worldwide [3,4,5]. The true incidence of CFP is difficult to ascertain due to under-reporting of CFP-related symptoms (2 to 10%) to health authorities [6]. These symptoms are characterized by gastrointestinal, neurological, and cardiovascular disorders, which often persist for days, months, or even years, and in some severe cases, can lead to paralysis and coma, and in extreme cases, even death [4,7]. 

More than 400 species of fish have been identified as vectors of CTXs [5]. The primary causative organism is the benthic dinoflagellate *Gambierdiscus toxicus* [1,5,8,9,10,11]. The genus *Gambierdiscus* comprises 15 species and 12 of them are toxic [11,12,13,14,15,16,17,18,19,20,21]. Other benthic dinoflagellate species of the genus *Ostreopsis* produce Palytoxin-like compounds (PTX) and some *Prorocentrum* species produce okadaic acid and its analogs (diarrheic shellfish toxins), which have also been associated with ciguatera [5,22]. However, there is little evidence that *Ostreopsis* and *Prorocentrum* species produce this syndrome [23]. Historical records of ciguatera have been documented in Mexico since 1862 [24]. In this study, we review the status of ciguatera in Mexico (epidemiological and treatment) from 1984 to 2013, the fish vectors implicated, and the potential causative dinoflagellates (*Gambierdiscus* spp., *Ostreopsis* spp., and *Prorocentrum* spp.). 

### 1.1. Historical Records

Before 1984, only two cases of ciguatera poisoning were reported in Mexico. In 1968, Halstead [24] described an episode that occurred in 1862 when a French crew sailing into the Gulf of Mexico became intoxicated after eating parrotfish (*Scarus* sp.). The same author also described another case that occurred in Puerto Vallarta, Jalisco in 1962 where intoxication occurred after consuming wahoo fish (*Acanthocybium solanderi*). 

### 1.2. Recent Cases

#### 1.2.1. Ciguatera in the Mexican Pacific

**Figure 1:**
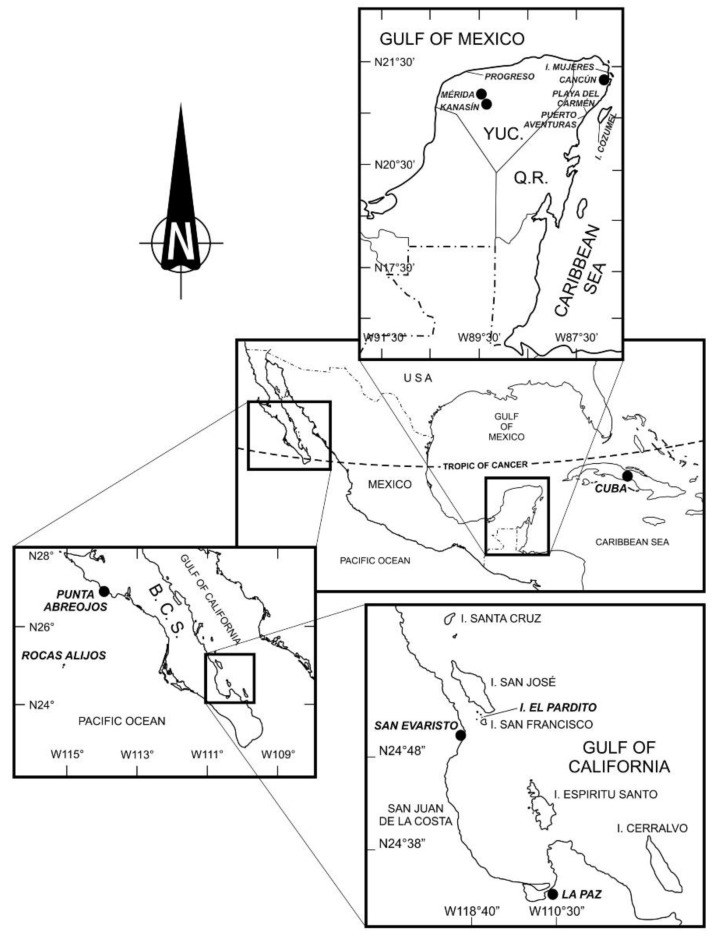
Locations of Ciguatera Fish Poisoning outbreaks along the Mexican coast: Rocas Alijos and Punta Abreojos in the Pacific Ocean; La Paz, San Evaristo, and El Pardito island in the Gulf of California; Mérida, El Progreso, and Kanasin in the Gulf of Mexico; and Isla Mujeres, Cozumel, Playa del Carmen, Puerto Aventuras, and Cancun in the Caribbean Sea.

All ciguatera cases registered in this area were reported in the state of Baja California Sur (B.C.S.). The cases were rare and sporadic and only four human intoxications have been reported from 1984 to 2011, involving a total of 240 people (Table 1) [25,26,27,28]. Parrilla-Cerrillo et al. [29] were the first to describe ciguatera in this region. In 1984, 200 persons became ill in La Paz, Baja California Sur (24.1405° N, 110.3123° W, Figure 1). The toxicity of snapper fish muscle of the genus *Lutjanus* was assessed by mouse bioassay (MBA) (Table 1 and Table 2). In 1992, 25 cases of CFP were associated with the consumption of flag cabrilla or starry grouper (*Epinephelus labriformis*) from Rocas Alijos (300 km west of Magdalena Bay; 24°57′31′′ N, 115°44′59′′ W, Figure 1; Table 1 and Table 2) [30]. In 1993, Lechuga-Devéze and Sierra-Beltrán [31] recorded another CFP outbreak, where seven members of a tuna ship crew were intoxicated after consuming cabrilla and garropa (*Epinephelus* sp. and *Mycteroperca* sp., respectively). In this last study, the presence of CTX-type toxins in the consumed fish was confirmed by mouse bioassay MBA (Table 1 and Table 2).

Between 1993 and 1997, fisherman reported five intoxications following consumption of fish liver from Serranidae (grouper) and Lutjanidae (snapper) in El Pardito Island and Punta San Evaristo (24°35.0′ N, 110°49.6′ W, Figure 1). The symptoms were diarrhea; nausea; eye, limb, and abdominal pains; headache; numbness; vomiting; weakness; pruritus; hyperesthesia; fatigue; desquamation around lips, eyebrows, hands, and feet; lip and tongue paralysis; and, in one case, convulsions. These symptoms appeared from 4 to 12 h after fish consumption and lasted a few days (Table 1 and Table 2). All these events occurred between March and July: one case in 1993, two in 1994, one in 1996, and another one in 1997 [28,32]. In the same location, the presence of neurotoxins of the CTX type were confirmed in livers of *M. prionura* and *L. colorado* by MBA and HPLC analysis [28,32].

Three new intoxication cases with similar symptoms occurred in 2004, involving the consumption of liver of big grouper (*Ephinephelus* sp.) in Punta Abreojos, B.C.S. (26°43′38′′ N, 113°34′29′′ W, Figure 1). The symptoms of these intoxications were more severe and lingered for several days, causing neurologic effects, with symptoms being reactivated by fish consumption several months afterward (unpublished data).

#### 1.2.2. Ciguatera in the Mexican Atlantic (Gulf of Mexico and Caribbean)

Information compiled from databases provided by the Secretaría de Salud (SS; Mexican Health Ministry) and scientific literature, indicate that, between 1994 and 2013, 14 CFP events occurred in the Mexican Atlantic (Table 1 and Table 2). During these events, a total of 208 poisonings were reported. In the state of Quintana Roo, 88 cases were reported in Isla Mujeres, 20 on the island of Cozumel (21°13′ N, 86°43′ W), 14 in Cancun (21°09′38′′ N, 86°50′51′′ W), 5 in Puerto Aventuras (20°37′39′′ N, 87°04′52′′ W), 32 in Playa del Carmen (20°30′42′′ N, 87°14′3′′ W), and 4 in Tulum (20°37′39′′ N, 87°04′52′′ W) (Figure 1). In the state of Yucatan, 26 cases were reported in Merida (20°58′1.56′′ N, 89°37′25.68′′ W), 11 in El Progreso (20°56′ N, 89°34′ W), 6 in Kanasin, and 2 in a non-specified site (Figure 1). All cases were related with the consumption of *Sphyraena barracuda* (great barracuda). According to Arcila-Herrera et al. [33], ciguatera has always been present in this region. It is common to not register these poisonings and many are only known as anecdotal accounts. Although reported incidents have increased, CFP still remains poorly studied in Mexico. Sixteen other cases of Mexican tourists intoxicated by CFP were reported in Playa Girón, Cuba (22°13′ N, 81°10′ W, Table 1 and Table 2, Figure 1), and are included in this review (Table 2).

Arcila-Herrera et al. described the first record of CFP in the area of Isla Mujeres [33]. In July 1994, 10 people from two families were intoxicated after consuming grilled barracuda. The intoxicated people were transferred to Cancun, Quintana Roo, where six were hospitalized with acute symptoms. CFP symptoms started between 20 min and 12 h after fish ingestion, with diarrhea and cold hypersensitivity being the most common clinical symptoms, and cold-to-hot temperature reversal dysesthesia occurring in all cases. The evolution of the symptoms in the patients was tracked over the subsequent 34 months by the medical center in the city of Merida, Yucatan. One case presented itchiness after drinking low quantities of alcohol. Four people developed chronic to moderate symptoms, and one person had persistent arthralgia that prevented some mobility, although improved following administration of indomethacin.

A second case of CFP involved 30 French tourists in Isla Mujeres during the summer of 1995. They also consumed grilled barracuda (10–12 kg) in a local restaurant. Patients were treated and hospitalized in the Antipoison Center of Marseille in France to evaluate the efficiency of the CFP treatment with mannitol. All patients presented gastrointestinal disorders within 4–6 h after consumption of barracuda, also developing neurosensory disorders and diffuse pain during the flight back to France. They presented low plasma cholinesterase levels. All patients who experienced high severity symptoms upon admission into the hospital reported persistent intoxication symptoms that lasted between one and seven months after fish consumption. In six of these patients, symptoms re-occurred following subsequent ingestion of seafood and alcohol [34,35].

In 1996, a CFP intoxication outbreak following consumption of barracuda affected six residents of Kanasin and 15 residents of Merida, Yucatan. Ten of the cases from Merida were infants between one and four years old, with the primary symptoms being paresthesia and muscular spasms [36]. A fourth case occurred in 2000 in Progreso, Yucatan following consumption of barracuda. Ten adults and a four-year-old child were intoxicated, with symptoms including muscular contracture, abdominal and limb pain, vomiting, diaphoresis, hypersalivation, affected awareness, severe muscular spasms, mydriasis of the central pupil, bradyarrhythmia, and filiform pulse. The affected individuals were hospitalized in the Centro Médico Americano in Progreso, with the child being transferred to the pediatric emergency center of the Hospital General Agustin O’Horan in Merida, and treated with mannitol, which resulted in improvement [37].

Farstad and Chow [38] described a case of CFP in 2001, where a woman and a man became intoxicated in Cancun, Quintana Roo. Symptoms, beginning six hours after consuming barracuda, included vomiting, abdominal pain, and profuse watery diarrhea that decreased within 12 h. Several days after the first symptoms, both patients noticed bizarre facial and extremity paresthesia, as well as the peculiar sensation that cold food seemed hot, and hot drinks felt icy cold. The woman had swollen glands in the neck and a sore throat. The man was more seriously affected with headaches, malaise, as well as debilitating and burning numbness in the hands and feet. When exercising, the man experienced unbearable pruritus and severe pubic pain during ejaculation. Five weeks after the onset of symptoms the CFP diagnosis was confirmed, but since both parties improved dramatically, no treatment was prescribed. After 10 weeks both patients were symptom free.

In 2004, Keynan and Pottesman [39] from the Infectious Diseases Unit, Bnei-Zion Medical Center in Haifa, Israel, described another CFP case. A woman developed paresthesia of the right arm and legs after traveling to the Peninsula of Yucatan and Guatemala where she consumed seafood, but could not recall a specific fish consumed. The paresthesia spread to her face, affecting the entire left side. She also felt cold and had difficulty falling asleep. The symptoms continued for at least five weeks, consisting of pruritus, paresthesia, myalgia, fatigue, and sleepiness.

In the autumn of 2006 in Isla Mujeres, Quintana Roo, nine local residents displayed CFP symptoms after consuming barracuda and were hospitalized at the Jesús Kumate Rodríguez General Hospital. They suffered diarrhea and vomiting that led to severe dehydration. These symptoms worsened and they were transferred to the Hospital General of Cancun. The Mexican government health authorities of the Comisión de Fomento Sanitario prohibited the sale of barracuda after this outbreak. 

In August 2007, 13 cases with symptoms of CFP occurred in Cozumel, Quintana (Q.) Roo that required hospitalization. In October of the same year, five more cases were reported in the same locality. Patients were hospitalized in the Hospital General de Cozumel and the Instituto Mexicano del Seguro Social after consuming barracuda. While hospitalized, two patients had convulsions and one had difficulties speaking. In April 2009, 12 fishermen of Isla Mujeres, Q. Roo were admitted to the Hospital Integral with CFP symptoms after consuming barracuda with an estimated weight to 12–15 kg. After two hours, they experienced gastrointestinal disturbances with vomiting, chills, muscle and headache pain, lip and tongue numbness, dehydration, and subsequently weight loss. 

In March 2010, a CFP outbreak involving 12 patients occurred in Cancun, Q. Roo. All had abdominal pain, diarrhea, general discomfort, muscular pain, vomiting, paresthesia, and headaches, with two cases requiring hospitalization. Later in the same month, in Puerto Aventuras, Q. Roo, another CFP outbreak was recorded involving five people following consumption of barracuda. All patients experienced diarrhea, general discomfort, muscular pain, vomiting, and paresthesia, and one person was hospitalized. 

In April 2010 in the city of Merida, 11 people from two families were intoxicated by consumption of barracuda acquired in a local market. Another outbreak occurred in August 2011 in Playa del Carmen, Q. Roo. In this event 29 people were intoxicated by eating barracuda acquired in a local fish market. In some cases, hospitalization lasted two weeks.

At the end of August 2011 in Isla Mujeres, Q. Roo, 27 people from five countries (Philippines, England, Canada, Mexico, and U.S.A.) were intoxicated by consuming barracuda (*Sphyraena*) and cubera (*Lutjanus* sp.). All were hospitalized in Cancun (Hospital General Z-3, Hospital Regional 17, Hospital Amerimed, and Hospital Galenia).

Two cases were reported in Playa del Carmen in March 2012, and in Tulum in May of 2013, with three and four people, respectively. These cases were also associated with the consumption of barracuda. They entered the Hospital General de Playa del Carmen.

The main signs and symptoms of intoxication in these cases (Table 2) were gastrointestinal symptoms (nausea, vomiting, abdominal pain, and diarrhea), neurological symptoms (paresthesia, dysesthesia, and dyspnea), neuropsychological (depression, emotional lability, and fatigue) symptoms, and, in some cases, cardiovascular symptoms (bradycardia and hypotension). Gastrointestinal symptoms arose in the first few hours, followed by neurological and neuropsychological symptoms, which lasted for weeks, and in some cases months (Table 2). In one case of the Isla Mujeres intoxications [33], symptoms remained even after one year. It is possible that other chronic ciguatera cases with long-term symptoms exist in Mexico; however, most patients associated with CFP outbreaks in Mexico have not been tracked for chronic, long-lasting effects. 

In the majority of outbreaks (84%) of CFP in Mexico, treatment was supported by the use of calcium gluconate, analgesic, antidiarrheal, antihistaminic, anti-inflammatory, antiemetic, antibiotics, anxiolytic, and vitamin supplements. Since 1995, however, mannitol has been used by physicians (Table 2) in four outbreaks (16%) with rapid health recovery of the affected patients. 

In Mexico, the body temperature of patients has unfortunately not been documented in the majority of CFP cases during the acute phase of intoxication, though this is a useful physiological parameter, given that hypothermia (<36.5 °C) has been observed in both intoxicated humans [42,43] and in murine models [44,45]. Obtaining this basic physiological parameter could be useful in the future monitoring of ciguatera fish poisoning cases.

In the Mexican Pacific, fish species involved in CFP were from Serranidae and Lutjanidae families, whereas in the Mexican Caribbean, 100% of the outbreaks involved barracuda (*S. barracuda*) (Table 1). When comparing the signs and symptoms between the Pacific and the Atlantic coast, the cases of intoxication from the Pacific were characterized by the early presence of neurological signs and desquamation in two outbreaks (Table 2).

### 1.3. Dinoflagellate Species Associated with CFP

The presence of *G. toxicus* along the Caribbean and Gulf of Mexico coasts has been reported since the 1990s [46,47,48,49]. However, Litaker et al. [15], based on both molecular and morphological characterization, considered that *G. toxicus* was not present in these zones. Therefore, previous reports of the presence of this species in these regions [50,51,52,53,54] are not conclusive and could be misidentifications of other *Gambierdiscus* species, such as *G. carpenteri*, *G. carolinianus*, or *G. caribaeus* [55,56,57,58]. These species, together with *G. belizeanus*, have shown toxicity in the Caribbean [59,60,61]; therefore, they could potentially be the species responsible for CTXs production along the Caribbean coasts of Mexico.

In the Pacific, there are reports of *G. toxicus* in Pardito Island, Magdalena Bay (Gulf of California), and the coasts of Nayarit and Revillagigedo [62,63,64,65]. However, considering the increasing diversity of this genus and the difficulty in identifying these species based only on morphological features, it is necessary to perform toxicological and genetic studies to confirm the presence of *G. toxicus* in this zone. Thus, the causative agent of intoxication events in the Pacific still requires confirmation. In addition, a newly described *Gambierdiscus* species, which may be involved, has been reported for Bahía de la Paz [66].

**Figure 2:**
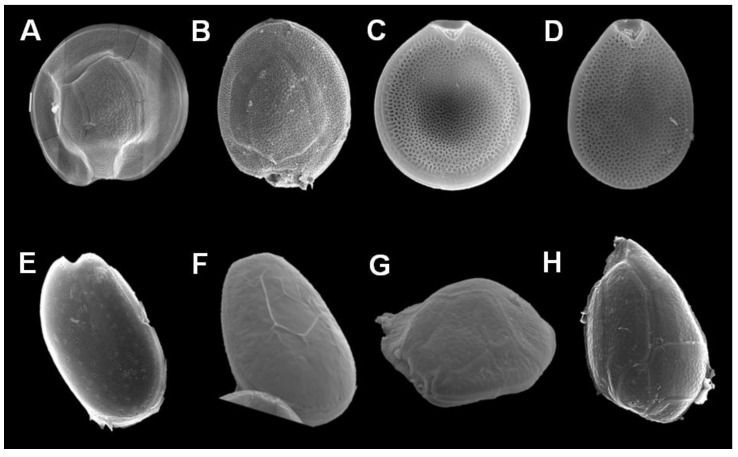
Scanning electron microscopy images of dinoflagellates potentially associated with ciguatera fish poisoning in Mexico. *Gambierdiscus* cf. *carolinianus* from the Mexican Caribbean: (**A**) epitheca, (**B**) hypotheca, and (**C**) *Prorocentrum belizeanus* from Puerto Morelos, Q. Roo; (**D**) *P. hoffmannianum* from Tulum, Q. Roo; (**E**) *P. lima* from Isla Contoy, Q. Roo; (**F**) *P. lima* (PRL-1) isolated from of El Pardito island, B.C.S.; (**G**) *O. siamensis* from Cabo Pulmo, B.C.S.; and (**H**) *O. heptagona* from Puerto Morelos, Q. Roo.

Increasing numbers of studies related to benthic dinoflagellates in the coasts of the Gulf of Mexico, Caribbean, Gulf of California, and Tropical Pacific of Mexico are being completed [51,52,54,67,68,69,70,71,72,73,74,75,76,77,78,79,80,81,82]. Consequently, the knowledge of the number of species and their geographic distribution has increased. At present, there is evidence for the occurrence of the following potentially toxic species: *Ostreopsis siamensis*, *O. lenticularis*, *O marina*, *O. ovata*, *Gambierdiscus* sp, *Prorocentrum lima*, and *P. rhathymum* along the Pacific coast (Table 3; Figure 2); and *G. carolinianus*, *G. belizeanum*, *G. caribaeus*, *G. carpenteri, Fukuyoa yasumotoi* (=*G. yasumotoi*), *Ostreopsis ovata*, *O. lenticularis, O. belizeanus*, *O. marina*, *O. siamensis, O. heptagona*, and several benthic species of the genus *Prorocentrum* (*P. lima*, *P. concavum*, *P. hoffmannianum, P. belizeanum, P. rhathymum* and *P. emarginatum)* along the Atlantic coast (Caribbean and Gulf of Mexico) (Table 3; Figure 2).

There are ongoing studies describing dinoflagellate distribution associated with ciguatera in Mexico. Several cultured strains of the dinoflagellate species mentioned above exist in national and international collections (Table 4). Morphological variation, molecular phylogeny, isozyme analyses, and toxicity of *Gambierdiscus* strains isolated from Mexican waters have been investigated in comparative studies between strains of different origins [46,59,60,61,83,84]. In most cases, the toxicity (toxin profile and toxin concentration) and the genetic and physiological characteristics of *Gambierdiscus* species reported in the literature are unknown. Most taxonomic and ecological studies have been performed with preserved samples.

Benthic dinoflagellates inhabit a variety of substrates. They have been collected from macroalgae, seagrasses, sandy bottoms, rocks, and coral skeletons in rocky and coral reefs [51,52,56,57,58,79,80,81,85]. *Ostreopsis siamensis* and *O. lenticularis* occur in the water column [78,82]. Demographic descriptions of benthic dinoflagellate communities are better known in the Atlantic than in the Pacific. *Prorocentrum* has the greatest species richness in the Caribbean and the Gulf of Mexico [51,56,79,80], and *P. lima* was reported as the dominant species in the port of Veracruz from March to June, reaching abundances of 2.97 × 10^4^ cells g^−1^ of *Thalassia testudinum* [51]. Along the Yucatan, *P. rhathymum* was the dominant species reaching similar abundances of 2.41 × 10^4^ cells g^−1^ [79,80]. In the Northern Caribbean, *Ostreopsis* (mainly *O. marina* and *O. heptagona*) was the dominant genus, reaching densities of 3.0 × 10^4^ cells g^−1^ [85]. *Gambierdiscus* genus has been registered in this zone but at abundances lower than 1 × 10^3^ cells g^−1^. 

These results suggest a high biodiversity, wide geographic distribution, and heterogeneous population dynamics of benthic dinoflagellates in the Mexican coastal waters, potentially explaining the unpredictability of CFP events. Ciguatera outbreaks could increase in the next few years along the coasts of Mexico due to the degradation of the coral reef and other coastal ecosystems (i.e., eutrophication).

### 1.4. Fish Species Involved in CFP

All recent cases of CFP along both the Pacific and Atlantic coasts of Mexico have been related to the consumption of carnivorous fish such as lutjanids (snapper), serranids (grouper), and sphryraenids (barracuda) (Table 5). These families of fish are commonly consumed and economically important in regional coastal fisheries, representing a valuable protein source for the population [87,88]. In the Caribbean, herbivorous fishes are not usually associated with ciguatera [89,90]. In contrast, many species of carnivorous fish cause ciguatera (Muraenidae, Lutjanidae, Serranidae, Lethrinidae, Scombridae, Carangidae, and Sphyraenidae). The last two families are especially important vectors of CFP in the Caribbean. 

### 1.5. Marine Toxins

#### 1.5.1. Dinoflagellate Toxins 

The toxicity of Mexican *Gambierdiscus* species has been evaluated in a few strains: CZ2, CZ3, and CZ4 (Table 4) isolated from Cozumel, Q. Roo (20°30’ N, 86°57’ W; Figure 1) and from the strains Mex Algae 1 gam, Mex Algae gam 1, and Mex Algae 2 gam 1 isolated from Cancun. Babinchak et al. [46] evaluated toxicity (CZ2, CZ3, and CZ4) by mouse bioassay, cytotoxicity assay in rat pituitary tumor cells (GH4C1), calcium uptake experiments, and the brevotoxin (PbTx) displacement assay; however, they did not describe the specific toxicity of the strains. Hemolytic activity (attributed to maitotoxin (MTX) of *G. carolinianus* Mex Algae gam 1 (149–201 EC_50_ cells log phase) and *G. caribaeus* Mexico Algae 1 gam 1 (198–212 EC_50_ cells log phase) was evaluated by in vitro human erythrocyte lysis assay [59]. Litaker et al. [61] analyzed the toxicity of *G. caribaeus* Mexico Algae 1 (1.29 ± 0.40 fg CTX3C eq. cell^−1^) and *G. carpenteri* Mexico Algae 2 Gam 1 (1.14 ± 0.18 fg CTX3C eq cell^−1^) in Cancun strains using a cell-based neuro-2a cytotoxicity assay. Heredia-Tapia et al. [75] evaluated the toxicity of *P. lima* (strain PRL-1) isolated offshore near Isla El Pardito in the Gulf of California (Figure 1) by mouse and *Artemia* bioassays, antimicrobial test, TLC, and HPLC-MS. Results demonstrated the presence of diarrhetic shellfish-toxins okadaic acid (OA) and dinophysistoxin-1 (DTX1) in the unusual proportion of 1:2, as well as a fast acting toxin (FAT), presumably prorocentrolide [91]. 

#### 1.5.2. Fish Toxins

**Figure 3:**
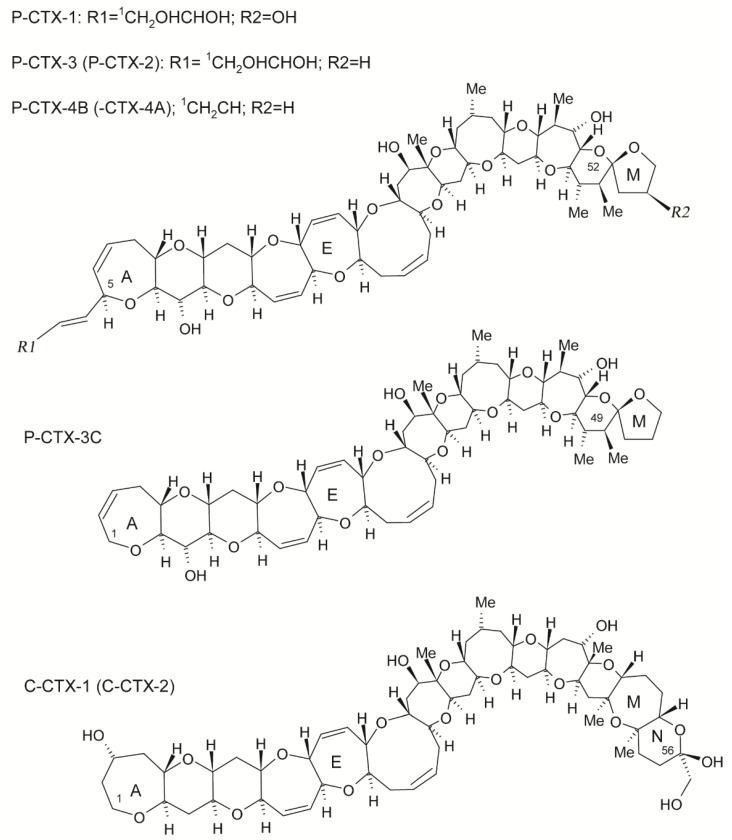
Structure of Pacific Ocean and Caribbean Sea ciguatoxins (CTXs): P-CTX-1 [93], PCTX-3 [94,95], P-CTX-4B [92], P-CTX-3C [96], and C-CTX-1 [97]. The energetically less-favored epimers, P-CTX-2 (52-epi P-CTX-3) [95], P-CTX-4A (52-epi P-CTX-4B) [98], and C-CTX-2 (56-epi C-CTX-1) [97], are shown in parenthesis. Image modified from Lewis. [90].

**Figure 4:**
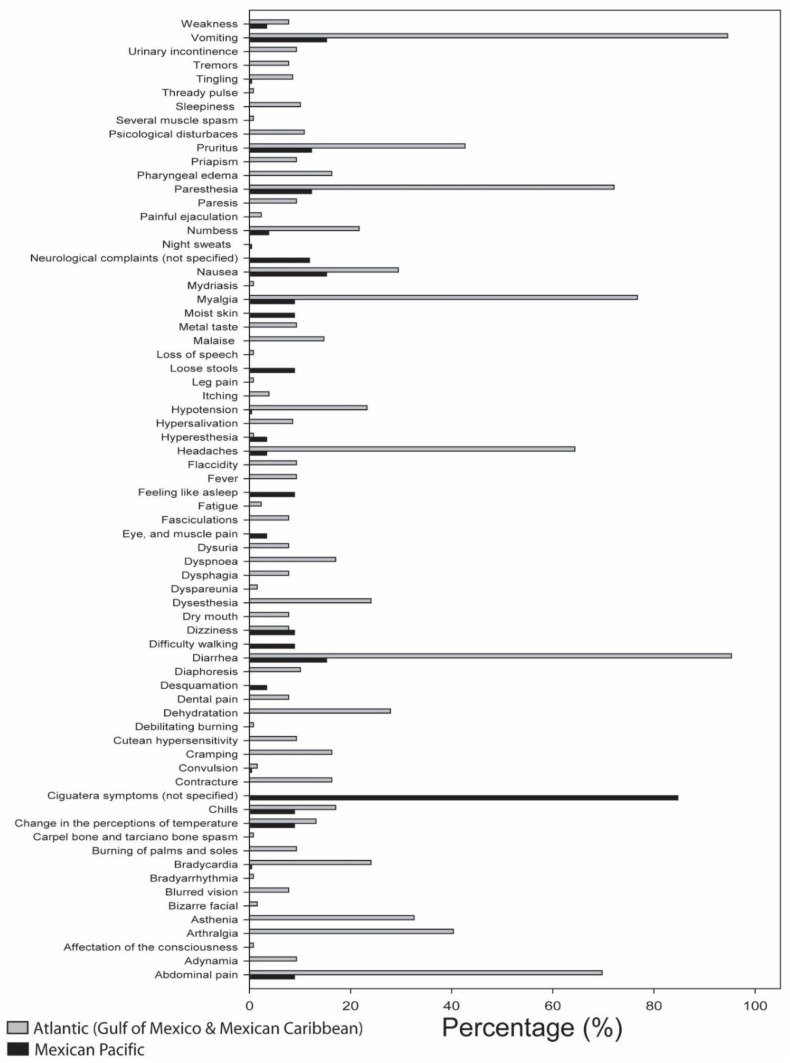
Frequency of symptoms (%) of ciguatera fish poisoning in Mexico (Atlantic and Pacific coasts).

Lewis [90] classified CTXs according to their area of origin: Pacific Ocean and Caribbean Sea CTX toxin families (Figure 3), with toxin structures differing between the two regions. In the Pacific zone, the most potent and principal ciguatoxin is ciguatoxin-1 (P-CTX-1; P stands for Pacific), followed by P-CTX-2 and P-CTX-3. Structural modifications were mainly observed in the terminal of the toxin molecules, mostly following oxidation. Caribbean ciguatoxins (C-CTX-1, C stands for Caribbean) are less polar than P-CTX-1 and Caribbean ciguatoxin structures (C-CTX-1 and C-CTX-2) have been determined [86]. Differences in the structure of the two families help explain the different symptoms of CFP in the Pacific and Caribbean regions [5]. Figure 4 compares the frequency of symptoms in CFP cases in the Mexican Pacific and Atlantic (Gulf of Mexico and Caribbean Sea).

Notably, in most of the Mexican CFP outbreaks, there has been no or minimal effort to determine the chemical nature and concentration of the toxins. Barton et al. [30] reported the first CFP epidemic involving 25 cases due to the consumption of flag cabrilla (*E. labriformias*). Hospitals in Southern California, U.S.A. successfully tracked the source of the outbreak to fish caught in the Rocas Alijos area off the coast of B.C.S., Mexico. Fish and blood samples of patients were sent to the University of Hawaii (Dr. Hokama Laboratory, Honolulu, HI, USA). A CTX-like compound was detected in the fish samples with a stick-enzyme immunoassay, although CTXs in blood samples from the affected patients were not determined, since normal serum lipids interfered with the assay. In the second case, Núñez-Vázquez et al. [32] described human poisoning events at El Pardito Island and Punta San Evaristo in B.C.S. in the Gulf of California that occurred during the spring season between 1993 and 1997. Gastrointestinal, neurological and cardiovascular disorders followed the consumption of liver from large *Serranidae* and *Lutjanidae* fish. During the spring of 1996, an adult specimen of *M. prionura* and another of *L. colorado* were obtained from the area. Liposoluble toxins extracted from the liver were evaluated by MBA, with mice developing clinical signs attributable to CFP. The sample from *M. prionura* reached 3.42 µg of ciguatoxin/kg of tissue. The extracts were analyzed by HPLC and samples from toxic fish showed chromatography peaks that were absent in negative fish controls. Their chromatographic behavior corresponded to fractions described as Ciguatoxin-1 (presumably P-CTX-1). LC-MS analysis did not reveal the presence of OA or its analogs in these samples (personal communication with Prof. T. Yasumoto). Only one report described the presence of OA in barracuda implicated in CFP in the Caribbean [92]. P-CTX-1 is one of the most important toxins in carnivorous fishes due to its high relative concentration in tissues as well as its toxicity [4]. This toxin presents the highest toxic potential of all the CTXs [1]. P-CTX-1 and P-CTX-3 analogues are thought to be products of the oxidative metabolism (biotransformation) of P-CTX-4B by cytochrome enzymes in fish livers [23]. P-CTX-3 is hypothesized to be an intermediate in the metabolism of GTX-4B to P-CTX-1. In the bio-oxidation of GTX-4B to P-CTX, there is a 10-fold increase in toxicity [93]. The biotransformation commonly occurs in the liver of the fish and can be a part of the detoxification process. Specifically, in major carnivorous fishes, the biotransformation of CTX-s to more polar forms with lower potency can be a strategy that accelerates the depuration and/or detoxification of the CTX-s in fish [9,23].

Compared with CFP, which is caused by CTXs, intoxication by palytoxin in fish seems to be much more severe and often results in death [99,100,101,102,103]. In addition to some of the hallmark symptoms of CFP, patients intoxicated with palytoxin exhibit acute respiratory distress and muscle spasms, cyanosis, metal taste, renal failure (no urine produced), and elevated serum enzyme levels. In severe cases, patients die within 30 min to a few days [99,100,103]. Wiles et al. [104] found the toxin hemorrhagic and destructive to cardiovascular, kidney, gastrointestinal, and respiratory systems [101]. Diarrhea is also reported in human poisoning cases and is an important indicator of experimental palytoxicosis, as well as cyanosis and progressive paralysis [105]. In all cases of CFP analyzed in this study for Mexico, no clinical signs or symptomatology (Table 2) corresponded to palytoxicosis, and as far as it was possible to determine, PTX and its analogs did not provoke the reported effects. 

### 1.6. Ecological Risk

Coral reef bleaching has been associated with extreme natural events such as hurricanes, climatic global change, the El Niño phenomenon, tsunamis, seismic activity, and submarine volcanism [106]. Physical deterioration of coral reefs are related to human activities such as tourism, military operations, industry developments, dyke construction, wharfs, and channel dredging, among others. Eutrophication and disturbance of coral reefs are also thought to increase the risk of CFP outbreaks by providing a benthic substrate for dinoflagellate growth [4,107,108,109,110,111,112].

Along the peninsulas of Baja California and Yucatan where tourism activities have increased significantly along with high population growth, the presence of CFP has increased in the last few years. These changes in human behavior have caused a higher contribution of wastewater from the cities, increasing the organic load in the ocean, which, together with increasing agricultural and aquaculture activities in the peninsula of Yucatan (Table 6), have helped cause eutrophication in some coastal areas [113].

According to the Consejo Nacional de Población, in 1950 [115], there were 1.54 million inhabitants along the Mexican Pacific coastal zone, with the population subsequently increasing to 9.04 million in 1995. On the west coast of the Gulf of Mexico during the same period, the number of inhabitants increased from 828,000 to 3.25 million, while in the Mexican Caribbean, the population increased from 183,000 to 1.37 million inhabitants [116]. The INEGI records from 2005 reported a population of more than 3 million inhabitants with a high rate of population growth associated with the increase in tourism [114]. 

Quintana Roo, Yucatan, and Baja California Sur are highly vulnerable to hurricanes. In the Atlantic Ocean, hurricanes have increased in intensity during the last few decades, impacting the Caribbean Sea, where the largest coral reefs exist. From 1988 to 2007, this area was affected by seven H3 to H5 hurricanes (Saffir-Simpson scale) that wreaked havoc upon the coasts. From 1995 to 2005, this zone was affected by four huge hurricanes (Table 7), which devastated sea shores, provoking the break-up and death of large portions of coral reefs [117]. 

Along the Mexican Pacific Coast, there has also been a high incidence of hurricanes, with an increase on the Baja California Peninsula. Ten hurricanes (H1 or H2 categories) have impacted Baja California Sur in the last three decades. Around 12 to 16% of the hurricanes originating in the NW Pacific basin affected the Baja California Sur coasts [118] (Table 7). However, information regarding the effects of these hurricanes on the coral populations is lacking. 

Fragments of dead coral could be a substrate for different macroalgae species [108], as they can be colonized by epiphytic dinoflagellates such as *G. toxicus*. Bagnis [119] concluded that the presence of *G. toxicus* is scarce in healthy coral reefs, but its abundance can increase rapidly between filamentous and calcareous macroalgae established on impacted coral reefs. Kohler and Kohler [108] found that dead or bleached coral is used as a substrate by macroalgae in the British Virgin Islands and United States Virgin Island. These macroalgae contained the toxic epiphytic dinoflagellates *O. lenticularis*, *Prorocentrum concavum,* and *P. lima*. They also observed different herbivorous fishes that grazed on filamentous macroalgae that were prey for carnivorous fish, providing a route for accumulation of CTXs in the trophic pathways of tropical coral reefs. The authors concluded that when the substrate is available, as in the case of affected coral reefs, the increase in ciguatera events may occur. Yasumoto et al. [120] reported that dead coral surfaces are covered by filamentous and calcareous macroalgae, which are a suitable environment for colonization and proliferation of *G. toxicus*. Bomber et al. [121] suggested that the macroalga *Heterosiphonia gibbesii*, based on its morphology, flexibility, and surface area, is also a good substrate for *Gambierdiscus* species. These algae probably excrete metabolites and produce chelating substances that promote the growth of *Gambierdiscus* and other toxic dinoflagellates [121,122]. It is hypothesized that hurricanes affecting the Caribbean Sea and the Gulf of Mexico have resulted in the death and fragmentation of coral reefs, increasing the habitat for *Gambierdiscus* populations and increasing the risk of CFP. However, this has not been confirmed for the coastal waters of Mexico. 

Anthropogenic eutrophication in coastal zones of Southeastern Mexico is increasing. Agriculture, aquaculture, and tourism services have decreased the water quality in coastal zones of the Yucatan Peninsula [123,124]. The northern coast of the Yucatan Peninsula harbors a karstic aquifer with a highly permeable carbonated substrate. There, the discharges from human activities reaches the groundwater and becomes an important source of inorganic nutrients and dissolved organic matter. The residues generated by chicken and pig farms (Table 6) could potentially increase the input of nutrients, including nitrogen- and phosphorus-containing compounds, which in turn could favor the proliferation of toxic microalgae species when the aquifer discharges into the coastal waters. Smayda [125] found a correlation between continental nutrient loads and phytoplankton blooms. The detection of 15 harmful phytoplankton species in this area suggests a deterioration of the coastal marine environment [113]. This deterioration could benefit the growth of some macroalgae species, increasing the substrate for toxic epiphyte dinoflagellates [48].

In Baja California Sur, some of the ecosystems at risk are the biosphere reserves and natural protected zones, as well as Ramsar sites [126]. Many of these ecosystems are pristine, although there are zones close to these sites with increasing human population, aquaculture, and tourism activities, mainly in the Los Cabos corridor and Bahía de La Paz. The coastal zone of these regions is influenced by the marine currents of the Gulf of California and by the tropical surface waters of the Pacific Ocean [127,128,129]. Seasonal water flow changes the water column, which suppresses the accumulation of nutrients, although historical data show variations in the nitrate, phosphate, and silicate concentrations, attributed mainly to the increase in tourism and land use [130]. 

Temperature is another key environmental variable capable of changing the structure and function of coastal ecosystems, with elevated sea temperature being a factor associated with the presence and transmission of ciguatoxin in the marine web [131]. Anomalous temperatures have been recorded, for example, in the Pacific Ocean during the El Niño-Southern Oscillation (ENSO) events. Hales et al. [109] calculated a positive correlation between superficial increase in sea temperature associated with El Niño and ciguatera in the islands of the South Pacific. These authors established that high temperatures promote bleaching and death of the coral reefs, followed by the colonization of dead coral fragments by macroalgae, and subsequently, of *Gambierdiscus* populations that use the macroalgae as substrates. Therefore, the presence of *Gambierdiscus* and CFP could be indicators of natural disturbances or anthropogenic impacts on coral ecosystems. 

During the 1982–1983 ENSO, large extensions of coral became bleached in Central America [132]. A similar phenomenon of coral death was recorded for the strongest El Niño in 1998 in the Indian Ocean [133]. 

Lechuga-Devéze and Sierra-Beltrán [35] associated ciguatera cases in Rocas Alijos in the Mexican West Pacific with possible thermal anomalies caused by the ENSO of 1991–1993. In artificial microcosms of coral reefs, Goodlett et al. [134] recorded two blooms of *G. toxicus* caused by water temperature variations and by a massive bloom of the macroalgae *Dictyota* sp. and high concentrations of copper. *Dictyota* is known to be a good substrate for the growth of *G. toxicus*. Increases in the population and toxicity of the benthic dinoflagellate *Ostreopsis lenticularis* and the low proportion of *G. toxicus*, as well as the presence of the carnivorous predator *Sphyraena barracuda*, were associated with high superficial sea temperature [131,132].

At the National Park of Cabo Pulmo, Baja California Sur in the Gulf of California, record anomalies above 3 °C were measured during the 1997–1998 ENSO, which were responsible for the bleaching of 32.2% and death of 59.3% of the coral population [135]. In 1996, 1998, 2010, and 2011, there were massive losses of fishes in the area and the presence of potentially harmful benthic microalgal species of the genus *Ostreopsis* (Figure 3), *Gambierdiscus*, *Amphidinium*, *Prorocentrum, Coolia*, and *Lyngbya* [136]. Proliferations of toxic benthic microalgae such as *Lyngbya majuscula* were detected in the Caleta Balandra (La Paz Bay, Gulf of California) from the summer of 2010 to date. In 2011, after a bloom of these cyanobacteria, two swimmers who had direct contact presented with intense dermatitis in the legs and arms. Samples of *L. majuscula* and of the opistobranch *Stylocueilus striatus* (which feed specifically on *Lyngbya*) presented toxicity of lyngbyatoxins- and aplysiatoxins-like compounds, and paralytic shellfish toxins were detected by MBA and HPLC-FLD [137,138].

Petroleum platforms can also act like an artificial coral reef, offering substrate for macroalgae and *Gambierdiscus* populations. Villarreal et al. [112] recorded *Gambierdiscus* in the structures located in a the western part of the Gulf of Mexico and concluded that these platforms are a risk factor for expansion of ciguatera due to future increase in sea surface temperature linked to the invasion of tropical species [106]. Currently, there are 255 platforms associated with the petroleum industry in the Mexican portion of the Gulf of Mexico [139]. These represent potential sites for the establishment of *Gambierdiscus* and other toxic microalgae species reported in the southeast of the Gulf of Mexico [47,48]. 

There are important zones with coral reefs in the southeastern side of the Gulf of Mexico where different epiphytic toxic microalgae species occur [47]. These reefs have been mainly affected by hurricanes and grounded ships associated with the petroleum, fishing, and tourism industries [116,140]. In the Sonda of Campeche, the Alacranes reef is one of the most affected areas. Since 1524, ships have been stranded in this area, and during the 1990s, more than five ships have grounded in this region [140] (Table 2). From 1990 to 2001, the coral reef in the state of Veracruz has been one the most affected by the severe impacts of ship groundings [141]. The damage to these reefs has also been associated with contaminants generated by the petroleum industry, river discharge, and human and agricultural residues. If physical deterioration of coral reefs continues, as well as thermal changes, water turbidity, hurricanes, construction of docks, piers, and industrial and tourist activities, there is a high probability that changes in the ecological equilibrium will favor the proliferation of toxic benthic species. On a positive note, the threats are now recognized and there are at least incipient management plans for these areas [142].

### 1.7. Legislation, Management, and Prevention

**Figure 5:**
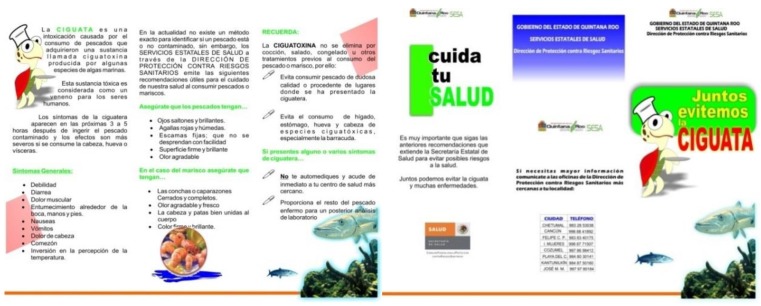
Example of educational material development for “Ciguata” (Ciguatera) (COFEPRIS, Quintana Roo, Mexico).

National and international sanitary legislations regulate CTXs. In some areas, public health officials have adopted actions to prevent CFP, like the prohibition of the commercialization of fish of some sizes or high-risk species fished from high-CFP-risk zones. The amount of the toxin accumulated in fish is thought, by many authors, to be related to their size and age. Consequently, when an intoxication case occurs, restrictions on fish sale are often imposed, in some cases without analyzing the presence of the toxins. Prohibitions of this type have been imposed in Samoa Americana, Queensland Australia, French Polynesia, Fiji, Hawaii, and Miami [5,143]. The United States Food Drug Administration has established concentration limits for natural CFP-related toxins as follows: 0.01 ppb P-CTX-1 equivalents for Pacific ciguatoxin and 0.1 ppb C-CTX-1 equivalents for Caribbean ciguatoxin [144]. In the European community, the Directiva del Consejo 91/493/EEC prohibits marketing marine products related to ciguatera. In Platypus Bay and Queensland, Australia, the capture of *Scomberomorus commerson* and *S. jello* is prohibited. Carnivorous fishes, such as those from the Muraenidae family and *Symphorus nematophorus*, among others, are not commercialized. In Mexico, the Comisión Federal para la Protección Contra Riesgos Sanitarios (COFEPRIS) [145] of the Secretaría de Salud published a recommendation of maximum values permissible for marine products of 2.5 MU/100 g of ciguatoxins [146]. Since 2007 in the Mexican Caribbean (Q. Roo), COFEPRIS took action to protect against health risks by CFP through the inspection of fishing cooperatives to restrict the capture and sale of fish species involved, particularly barracudas. They implemented an inspection of seafood product retail establishments and, in some instances, destroyed fishery products from species considered to be hazardous to public health. Additionally, they implemented a public information campaign through the distribution of 8,000 leaflets (Figure 5) with the theme “Together we avoid Ciguatera” [147].

## 2. Conclusions

There have been 464 CFP intoxication cases over 25 separate outbreaks recorded in Mexican coastal zones between 1984 and 2013: 240 (51.72%) in Baja California Sur, 163 (35.12%) in Quintana Roo, 45 (9.69%) in Yucatan, and 16 (3.44%) cases of Mexican tourists intoxicated in Cuba (Table 1). Carnivorous fish were involved in all cases: snapper (*Lutjanus*) and grouper (*Epinephelus* and *Mycteroperca*) in the Pacific Ocean, and snapper (*Lutjanus*) and great barracuda (*S. barracuda*) in the Atlantic Ocean (Gulf of Mexico and Caribbean Sea) (Table 5). However, an underestimation of CFP cases in the Mexican Caribbean is likely, since this problem is likely to have always been present. Reports of CFP in Mexico are increasing, although they have been poorly studied. Present records suggest that CFP is the second most common cause of seafood intoxication in Mexico, preceded only by paralytic shellfish poisoning. The dinoflagellates that have been linked to CFP events belong to the genus *Gambierdiscus*, *Ostreopsis*, and *Prorocentrum* (Table 3 and Table 4; Figure 3). However, the toxicity (toxins profile and levels), physiology, and genetic identification of most of the dinoflagellates species is unknown. The chemistry of the toxins s has only been determined in fish samples by Barton et al. [30] and Núñez-Vázquez et al. [32], so considerably more work is required to elucidate the toxins present in Mexican CFP outbreak samples.

The incidence and propagation of *Gambierdiscus* species is associated with bleaching and death of coral reefs provoked by the changes in sea temperature and the fragmentation of reefs caused by hurricanes. Also, anthropogenic activities, such as petroleum platforms, marine construction, and other tourism developments, create substrates for different species of macroalgae, microalgae, and benthic invertebrates, which might influence toxic dinoflagellate populations. In Mexico, there is limited research on the physiology and ecology of *Gambierdiscus* and other toxic benthic species, so developments in this area are required. The peninsulas of Yucatán and Baja California Sur, which are regions where ciguatera has been recorded, could provide a good baseline for ciguatera studies associated with anthropogenic effects and natural phenomenon, since multiple activities are steadily increasing in both zones, such as aquaculture, infrastructure development, tourism, and human population growth. Additionally, these zones are frequently affected by hurricanes. It is important to have a continuous monitoring program in this area with a permanent site to monitor the toxicity and the factors that favor the proliferation and production of *Gambierdiscus*/CTXs [47].

Mexico is surrounded by four seas (Pacific Ocean, Gulf of California, Gulf of Mexico, and Caribbean Sea) with a high number of endemic species, species richness, and diversity, which are comparable to its continental biota. Marine and land biodiversity are complementary, having different ecosystems and biota that confer upon Mexico the designation of the fourth-most megadiverse country in the world [148]. This megadiversity is attributed to Mexican marine microorganisms and possibly to metabolites (phycotoxins included) they produce (chemical diversity), making it necessary to develop a knowledge database of the responsible organisms to a species level, as well as genotypes. This should include information of the environmental conditions that favor the growth of these species and CTX prevalence and composition in each case. 

The existing research highlights the importance of performing systematic investigations following reported poisoning cases after seafood consumption in Mexico. There is an absence of comprehensive and reliable epidemiology records of CFP cases in Mexico, which may have resulted from a shortage of human resources with the knowledge for accurate diagnostics of all cases. With the increased number of cases and expected continuation in future decades, there is an urgent need to identify the toxicity levels (periodicity and anatomical distribution) and to determine the toxin chemistry, as well as the toxin biotransformation products, in affected fish and fish consumers in order to develop and ultimately deliver effective treatment.

## Figures and Tables

**Table 1:** Events of ciguatera fish poisoning in Mexico (1984–2013).

Cases (M:F)	Locality (Cases)	Fish Involved	Analysis	Year	Reference
200 (n.d.)	La Paz, B.C.S.	*Lutjanus* sp.	MBA	1984	[29]
25 (17:4) *	Rocas Alijos, B.C.S.	*Epinephelus labriformis*	MBA and immunoassay	1992	[30]
7 (7:0)	Rocas Alijos, B.C.S.	*Epinephelus* sp. and *Mycteroperca* sp.	MBA	1993	[31]
5 (4:1)	Isla El Pardito (3), Punta San Evaristo (2), B.C.S.	*Mycteroperca prionura* and *Lutjanus colorado*	MBA and HPLC	1993–1997	[32]
3 (1:3)	Punta Abreojos, B.C.S.	*Epinephelus* sp.	MD	2004	[28]
10 (5:5)	Isla Mujeres, Q. Roo	*Sphyraena barracuda*	MD	1994	[33]
30 (14:16)	Isla Mujeres, Q. Roo	*S. barracuda*	MD	1995	[34,35]
21 (n.d.)	Merida (15), Kanasin (6), Yuc.	*S. barracuda*	MD	1996	[36]
11 (1:0) *	Progreso, Yuc.	*S. barracuda*	MD	2000	[37]
2 (1:1)	Cancun, Q. Roo	*S. barracuda*	MD	2001	[38]
1 (0:1)	Yucatan	n.d.	MD	2004	[39]
9 (n.d.)	Isla Mujeres, Q. Roo	*S. barracuda*	MD	2006	HMR
13 (n.d.)	Cozumel, Q. Roo	*S. barracuda*	MD	2007	HMR
5 (n.d.)	Cozumel, Q. Roo	*S. barracuda*	MD	2007	HMR
1 (0:1)	Mexico	n.d.	MD	2008	[40]
2 (n.d.)	Cozumel, Q. Roo	*S. barracuda*	MD	2009	HMR
12 (12:0)	Isla Mujeres, Q. Roo	*S. barracuda*	MD	2009	HMR
11 (n.d.)	Merida, Yuc.	*S. barracuda*	MD and MBA	2010	HMR
12 (4:8)	Cancun, Q. Roo	*S. barracuda*	MD	2010	HMR
5 (2:3)	Puerto Aventuras, Q. Roo	*S. barracuda*	MD	2010	HMR
29 (n.d.)	Playa del Carmen, Q. Roo	*S. barracuda*	MD	2011	HMR
27 (14:13)	Isla Mujeres, Q. Roo	*Lutjanus* sp. & *S. barracuda*	MD	2011	HMR
3 (2:1)	Playa del Carmen, Q. Roo	*S. barracuda*	MD	2012	HMR
4 (3:0)	Tulum, Q. Roo	*S. barracuda*	MD	2013	HMR
16 (3:9)*	Cuba	*Lutjanus* sp.	MD	1986	[41]
Total: 464 (90:66)					

* Incomplete information of male:female (M:F) cases ** Personal communication Dr. F. Chávez-Peón (Centro Nacional de información y Documentación de Salud, Secretaría de Salud). n.d. (not determined). MBA: Mouse bioassay. CTX presence was determined using a refined bioassay method based on Lewis et al [31,32]). MD: Medical Diagnostic. HMR: Health Ministry Report. Q. Roo=Quintana Roo. Yuc. = Yucatan.

**Table 2:** Signs, symptoms, and treatment of CFP cases in Mexico (1984–2013).

Locality/Year	Signs & Symptoms	Duration	Treatment	Ref.
La Paz, B.C.S., 1984	Ciguatera symptoms (not specified).	n.d.	n.d.	[29]
Rocas Alijos, B.C.S., 1992	Nausea; vomiting; diarrhea; loose stools; neurological complaints; paresthesia of the face, arms, and legs; bradycardia and hypotension; dizziness; stomachache; tingling and numbness in legs and fingers, feeling like they were asleep; moist skin; difficulty walking; myalgia; chills; sensitive cold; temperature reverse; pruritus; and night sweats.	Several days	Histamine blockers and palliative supports	[30]
Rocas Alijos, B.C.S., 1993	Diarrhea, nausea, vomiting, neurophysiological disorders.	15 days	n.d.	[31]
El Pardito Island and Punta San Evaristo B.C.S., 1993–1997	Diarrhea, nausea, vomiting, eye; muscle and abdominal pain; headache, numbness, weakness, pruritus, desquamation, hyperesthesia, lip and tongue paralysis, and convulsion in one case.	Several days	Palliative support, histamine blockers, and antibiotics	[28,32]
Isla Mujeres, Q. Roo, 1994	Gastrointestinal disturbances, watery diarrhea (dehydration and shock in 2 cases), cold-to-hot temperature reversal, dysesthesia in all cases with differences in the occurrence of nausea, vomiting, cramps, abdominal pain, weakness, paresthesia, arthralgia, myalgia, dizziness, dysuria, dyspnea, headache, pruritus, lip numbness, dry mouth, dental pain, chills, tremors, fasciculations, blurred vision, hypersalivation and dysphagia, emotional lability (2 cases). Painful ejaculation and dyspareunia (2 cases). Nipple hyperesthesia (1 female).	Chronic (4 cases, several months)	Palliative supports. Indomethacin (1 chronic case)	[33]
Isla Mujeres, Q. Roo, 1995	Hypotension, bradycardia, headache, arthralgia, pruritus, myalgia, asthenia, paresthesia in tongue, lips and extremity, abdominal pain, vomiting, diarrhea.	1–7 months	Antidiarrheal drugs, vitamins C, B1, B6 and B12, anti-histaminics, anxiolytic drugs, atropine, mannitol	[34,35]
Merida and Kanasin, Yuc., 1996	Paresthesia and muscle spasm, pharyngeal edema dysesthesia, contracture.	n.d.	Palliative supports	[36]
Merida, Yuc., 2000	Abdominal and leg pain; vomiting; muscle contracture; diaphoresis; hypersalivation; severe muscle, carpel bone and tarciano bone spasm; mydriasis, bradyarrhythmia; bradycardia; thready pulse; awareness alteration (1 child).	Days	methylprednisolone, diazepam, adrenaline, atropine, calcium gluconate, mannitol	[37]
Cancun, Q. Roo, 2001	Vomiting, abdominal pain, profuse watery diarrhea, fatigue, bizarre facial and extremity paresthesia, as well as the peculiar sensation that cold foods seemed hot, and hot drinks tasted ice cold, headaches, malaise, and debilitating burning and numbness in hand and feet, pruritus. During ejaculation, severe pubic pain.	Several weeks	n.d.	[38]
Yucatan, 2004	Paresthesia extended from face to hands and legs, muscle pain, pruritus, myalgia, fatigue, sleepiness.	Several weeks	Clarithromycin, ibuprofen	[39]
Isla Mujeres, Q. Roo, 2006	Diarrhea, vomiting, severe electrolyte imbalance.	Days	Palliative supports, serum	HMR
Cozumel, Q. Roo, 2007	Diarrhea, vomiting, dehydration.	Days	Palliative supports, serum	HMR
Cozumel, Q. Roo, 2007	Diarrhea, muscle pain, nausea, vomiting, headache, itching; change in temperature perception, convulsion (two cases), speech loss (1 case), mouth, hand and feet numbness.	Days	Palliative supports	HMR
Mexico n.d.	Headache, pain in back and joints, abdominal discomfort and sweating, nausea and itching.	Days	Palliative supports	[40]
Cozumel, Q. Roo, 2009	Diarrhea and dehydration.	Days	Palliative supports	HMR
Isla Mujeres, Q. Roo, 2009	Abdominal pain, headache, diarrhea, vomiting, chills, fever, muscle pain, numbness of limbs and tongue, dehydration.	Days	Palliative supports, serum	HMR
Merida, Yuc., 2010	Abdominal pain, diarrhea, nausea, vomiting, cramps, tingling, muscle pain.	Days	Palliative supports	HMR
Cancun, Q. Roo, 2010	Abdominal and muscle pain, diarrhea, vomiting, paresthesia, headache, general discomfort.	Days	Palliative supports	HMR
Puerto Aventuras, Q. Roo, 2010	Diarrhea, vomiting, muscle pain, paresthesia, general discomfort.	Days	Palliative supports	HMR
Playa del Carmen Q. Roo, 2011	Diarrhea, nausea, vomiting, dehydration, muscle pain, paresthesia.	Days	Palliative supports	HMR
Isla Mujeres, Q. Roo, 2011	Diarrhea, nausea, vomiting, abdominal pain, paresthesia.	Days	Hartmann solution, hydrocortisone, mannitol and adrenaline	HMR
Playa del Carmen, Q. Roo, 2012	Profuse watery diarrhea, vomiting, colic, paresthesia extended from face to hands and legs, hypothermia sensation.	Days to weeks	Palliative supports	HMR
Tulum, Q. Roo, 2013	Diarrhea, abdominal pain, paresthesia, respiration difficulty, weakness.	Days	Mannitol	HMR
Cuba, 1986	Profuse watery diarrhea, abdominal pain, nausea, vomiting, metal taste, diaphoresis, headache, myalgia, arthralgia, paresthesia, paresis, flaccidity, asthenia, adynamia, dyspnea, skin hypersensitivity, burning of palms and soles, intense pruritus, priapism, urinary incontinence, sleepiness, affliction, depression.	Months	Antihistaminic, antiemetic, analgesic, tranquillizer, palliative supports	[41]

HMR: Health Ministry Report.

**Table 3:** Distribution of potentially toxic dinoflagellates along Mexican coasts.

Dinoflagellate	Distribution	Reference
*Gambierdiscus toxicus*	Quintana Roo, Yucatán, Tabasco, Veracruz, Nayarit (Isla Isabel), B.C.S., Revillagigedo Islands	[46,47,48,49,50,51,52,53,63,64,65]
*G. carolinianus*	Quintana Roo	[57,58,59,61]
*G. belizeanum*	Quintana Roo	[49,50,58]
*G. caribaeus*	Quintana Roo, Yucatán	[56,57,58,59,80,85]
*G. carpenteri*	Quintana Roo	[61]
*Gambierdiscus* sp.	Campeche, Baja California Sur	[54,66]
*F. yasumotoi (=G. yasumotoi)*	Quintana Roo	[53,54]
*Ostreopsis ovata*	Baja California, Baja California Sur	[25,63,86]
*O. lenticularis*	Baja California Sur, Revillagigedo Islands	[63,82]
*O. marina*	Quintana Roo, Baja California Sur	[81,85,86]
*O. belizeanum*	Quintana Roo	[81]
*O. siamensis*	Quintana Roo, Nayarit (Isla Isabel), B.C.S. (Isla San José), Revillagigedo Islands	[57,59,63,66,77]
*Ostreopsis* sp.	Revillagigedo Islands, B.C.S.	[78]
*O. heptagona*	Veracruz, Quintana Roo	[51,57,79,80,81,85]
*P. lima*	Quintana Roo, Yucatán, Veracruz, Baja California, Baja California Sur, Sonora, Oaxaca	[25,49,51,56,57,63,64,66,71,73,75,79,80,85,86]
*P. hoffmannianum*	Quintana Roo, Campeche, Yucatán, Veracruz	[49,51,54,56,57,79,80,85]
*P. concavum*	Quintana Roo, Yucatán, Veracruz, Baja California Sur	[25,51,56,57,63,79,86]
*P. belizeanum*	Quintana Roo, Yucatán, Baja California Sur	[49,57,63,64,79,85]
*P. rhathymum*	Quintana Roo, Yucatán, Baja California Sur	[49,56,57,63,66,79,80,85]

**Table 4:** Species and strains of benthic dinoflagellates potentially associated with ciguatera fish poisoning in Mexico.

Dinoflagellate	Strain	Origin	Culture Collection	Reference
*G. toxicus*	CM2K, CM3K & CM4KI M510K, IM512K IM513K & IM514K CM515K, reef CM516K, reef CM517K, reef CM518K, reef CM519K, reef CM520K, reef	Cozumel, Q. Roo Parque El Garrafón, Isla MujeresClub Med, Cancun, Q. Roo	Tropical Dinoflagellate (Southern Illinois University, Carbondale)	[47]
	PO528K, Lagoon CZ2, CZ3 & CZ4	Pat O’Brien’s, Cancun, Q. Roo Cozumel, Q. Roo	National Marine Fisheries Service (Charleston Lab.)	[46]
*G. caribaeus*	Mex Algae 1 Gam 1	Cancun, Q. Roo		[59]
*G. carpenteri*	Mex Algae 2 Gam 1	Cancun, Q. Roo		[61]
*G. carolinianus*	Mex Algae Gam 1	Cancun, Q. Roo		[59]
*P. lima*	CM563K, reef PRL-1 PLV-1,a PLV-3	Club Med, Cancun, Q. Roo Isla El Pardito, B.C.S. B. de La Paz, B.C.S.	Tropical Dinoflagellate CODIMAR-CIBNOR CODIMAR-CIBNOR	[47] [86] [86]
*P. belizeanum*	CM559K, reef CM560K, reef CM561K, reef CM568K, reef IM553K CZ565	Club Med, Cancun, Q. Roo Parque El Garrafón, Isla Mujeres, Q. Roo Chankanaab reef, Cozumel	Tropical Dinoflagellate	[47]
*P. concavum* *P.* cf. *concavum*	CM559, reef PCJV-1 a PCJV-3	Club Med, Cancun, Q. Roo Isla San José, B.C.S.	Tropical Dinoflagellate CODIMAR-CIBNOR	[47] [86]
*P. mexicanum*	PO567AK, lagoon PXCV-1, PXPV-1 & PXPV-2	Pat O’Brien’s, Cancun, Q. Roo B. Concepción & B. de La Paz, B.C.S.	Tropical Dinoflagellate CODIMAR-CIBNOR	[47] [86]
*O. marina*	OMPV-1	B. de La Paz, B.C.S.	CODIMAR-CIBNOR	[86]
*O.* cf. *ovata*	OOJV-1 a OOJV-9 OOPV-1	Isla San José, B.C.S. B. de La Paz, B.C.S.	CODIMAR-CIBNOR	[86]
*Ostreopsis* sp.		San José del Cabo, B. C. S	Strain Collection of the CIBNOR	Virgen-Félix, 2008 *

* Personal communication with M. Virgen-Félix (responsible for the strain collection of CIBNOR). CODIMAR=Colección de Dinoflagelados Marinos del CIBNOR.

**Table 5:** Fish species responsible for ciguatera fish poisoning in Mexico (1984–2013).

Responsible Fish Genera	Frequency	Percentage	Intoxications (%)
*Lutjanus*	4	14.28	48.81
*Epinephelus*	3	10.71	6.88
*Mycteroperca*	2	7.14	2. 36
*Sphyraena*	17	60.71	41.53
Unknown	2	7.14	0.39
Total:	28	100	100

**Table 6:** Data of population, hotels and other socio-economic activities in Quintana Roo, Yucatán, and Baja California Sur, Mexico. Mexican states with the presence of ciguatera fish poisoning.

Data	Quintana Roo	Yucatán	BCS
Inhabitants	1,215,237	1,818,948	535,808
Hotels	763	330	290
Hotel rooms	73,108	8880	15 384
Tourists	7,546,720	1,589,940	1,834,515
Touristic docks	14	12	7
Biosphere reserves	3	2	2
National parks	6	2	2
Water treatments plants	29	260	94
Volume of water discharged under federal control (million m^3^)	215,193,50	51.0	1279.0
Fisheries production (ton)	3861	27,179.2	134,803
Aquaculture production (ton)	56.79	73.4	4421
Swine production (heads)	156,375	792,202	15,210
Avian production (heads)	4,003,730	17,512,206	62,087

Source: Instituto Nacional de Estadística, Geografía e Informática (INEGI), 2005–2007 [114].

**Table 7:** Hurricanes with ground impact in Quintana Roo, Yucatan, and Baja California Sur, Mexico. The sites of impact are cities where ciguatera fish poisoning had been documented.

Year/Hurricane	Site of Impact	Wind Intensity (km/h)	Category Saffir-Simpson
Gulf of Mexico and Caribbean Sea			
1988 Gilbert	Quintana Roo	287	H5
	Yucatan		
1995 Roxana	Tulum, Q. Roo	185	H3
1996 Doly	Felipe Carrillo Puerto, Q. Roo	130	H1
2000 Keith	Chetumal, Q. Roo	148	H1
2002 Isidore	Telchac Puerto, Yucatan	205	H3
2005 Wilma	Isla Cozumel, Q. Roo	230	H4
	Puerto Morelos, Q. Roo	220	H4
2005 Emily	Tulum, Q. Roo	215	H4
2005 Stan	Felipe Carrillo Puerto, Q. Roo	75	Tropical Storm
2007 Dean	Quintana Roo	280	H4
	Yucatan		
2008 Dolly	Cancun, Q. Roo	85	Tropical Storm
Pacific Ocean			
1995 Henriette	Cabo San Lucas, B.C.S.	158	H2
1996 Fausto	Todos Santos B.C.S.	130	H1
1997 Nora	Bahía Tortugas, B.C.S.	120	H1
1998 Isis	Los Cabos, B.C.S.	110	Tropical Storm
1999 Greg	San José del Cabo, B.C.S.	120	H1
2001 Juliette	La Paz, Ciudad Constitución, B.C.S.	120	H1
2003 Ignacio	Ciudad Constitución, B.C.S.	165	H2
2003 Marty	San José del Cabo, B.C.S.	160	H2
2006 John	El Saucito, B.C.S.	175	H2
2007 Henriette	Los Cabos, B.C.S.	150	H1
2008 Norbert	Bahía Magdalena, B.C.S.	215	H2

Sources: CNA, Estadísticas del agua en Mexico, 2007. Comisión Nacional del Agua. Mexico, D. F., 2007.
